# From overqualification to job crafting: how illegitimate tasks and moral distress shape physicians’ self-regulation

**DOI:** 10.3389/fpsyg.2026.1800281

**Published:** 2026-05-28

**Authors:** Tongyao Li

**Affiliations:** Department of Management, Sichuan Agricultural University, Chengdu, China

**Keywords:** calling (presence), illegitimate tasks, job crafting, moral distress, perceived overqualification, professional identity

## Abstract

Limited empirical research explains how physicians’ perceived overqualification translates into moral distress and divergent forms of job crafting when administrative duties are experienced as role-incongruent. I surveyed 437 practicing physicians in Chengdu, China. I estimated a moderated serial process model in structural equation modeling with robust maximum likelihood estimation. Perceived overqualification was associated with stronger perceptions of unreasonable, illegitimate tasks, which were linked to higher moral distress; moral distress, in turn, was associated with lower approach job crafting and higher avoidance job crafting. The indirect pathway from overqualification to job crafting through unreasonable tasks and moral distress was negative for approach crafting and positive for avoidance crafting. Calling intensified the association between unreasonable tasks and moral distress, and professional identity weakened the extent to which moral distress translated into reduced approach crafting. These findings integrate overqualification, illegitimate tasks, and moral distress within a single self-regulation process and clarify how identity conditions shape physicians’ coping through job crafting.

## 1 Introduction

Perceived overqualification refers to employees’ belief that their education, skills, experience, or abilities exceed the requirements of their current job (Maynard et al., 2006; Zhang et al., 2016). In most organizational research, this condition is treated as a form of person–job misfit that can reduce satisfaction, increase withdrawal, or motivate employees to restore fit through job crafting (Debus et al., 2020; Maynard et al., 2006; Woo, 2020). This framing is useful, but it is incomplete for clinical work. Physicians not only evaluate whether their abilities are fully used But they also evaluate whether assigned duties are compatible with professional expertise, ethical standards, and the responsible use of scarce clinical time.

In hospitals, surplus professional capability can become ethically consequential when specialized physicians are assigned unreasonable administrative or non-core duties. Such duties are not merely additional tasks. They can be interpreted as role-incongruent demands that signal the misallocation of expertise. Stress-as-Offense-to-Self theory suggests that illegitimate tasks are harmful because they communicate disrespect and violate role-based expectations (Semmer et al., 2019; Semmer et al., 2015). Moral distress scholarship further suggests that clinicians experience ethical strain when organizational conditions constrain action that would align with professional standards (Epstein et al., 2019; Morley et al., 2019). The central problem is therefore not whether overqualified physicians experience generic strain. The sharper question is how underused professional capability becomes a role-legitimacy problem that is associated with moral distress and different forms of self-regulation.

Job crafting provides the behavioral endpoint for this process because physicians may respond to misfit in different ways. Approach job crafting involves seeking resources and challenges, whereas avoidance job crafting involves reducing hindering demands (Lin et al., 2023; Petrou et al., 2012; Tims et al., 2012). Existing perceived overqualification research suggests that overqualification can be associated with both proactive and defensive responses (Fan and Shang, 2025; Shang et al., 2024). What remains unclear is why the same perceived surplus of capability may coexist with growth-oriented crafting and protection-oriented crafting in a high-stakes professional setting. I argue that the answer lies in a serial role-legitimacy process. Physicians who feel overqualified are more likely to appraise unreasonable non-core duties as illegitimate. These appraisals are linked to moral distress, which is then associated with lower approach crafting and higher avoidance crafting.

Prior studies leave three linked gaps. First, perceived overqualification studies have usually explained workplace outcomes through misfit, deprivation, boredom, entitlement, or self-view mechanisms, while giving less attention to ethical distress in professional work (Chang and Jiang, 2025; Fan and Shang, 2025; Ren et al., 2025; Shang et al., 2024). Second, illegitimate task studies show that unreasonable tasks violate role expectations, but they rarely position perceived overqualification as an upstream condition that makes role misallocation more salient (Fan et al., 2023; Ma and Xie, 2024). Third, moral distress research documents clinicians’ ethical strain and adverse well-being outcomes, but it rarely links moral distress to approach and avoidance forms of job crafting (Epstein et al., 2019; Lin et al., 2023; Morley et al., 2019). These gaps mean that existing research does not yet explain how surplus professional capability becomes an ethical self-regulation process in physicians’ work.

To address this problem, this study tests a moderated serial process model in which perceived overqualification is associated with unreasonable, illegitimate tasks, moral distress, and two forms of job crafting. The model also specifies calling and professional identity as identity conditions. Calling is expected to strengthen the association between unreasonable tasks and moral distress because strongly called physicians attach greater moral significance to professional work. Professional identity is expected to buffer the association between moral distress and job crafting because it anchors physicians to role-consistent action. The study is guided by four research questions.

- RQ1. How is perceived overqualification related to doctors’ job crafting behaviors, such as approach job crafting and avoidance job crafting?
- RQ2. How is perceived overqualification related to doctors’ perceptions of unreasonable illegitimate tasks, and how do these tasks relate to their moral distress?
- RQ3. Do unreasonable illegitimate tasks and moral distress sequentially explain how perceived overqualification is associated with doctors’ job crafting behaviors?
- RQ4. Under what conditions are these relationships stronger or weaker, specifically when doctors have a higher calling and a stronger professional identity?

This study makes four contributions. First, it extends perceived overqualification research by identifying a role-legitimacy pathway in clinical work. The contribution is not simply that overqualification is linked to strain. It is possible that overqualification may become ethically consequential when surplus clinical expertise is associated with the perception that assigned duties are unreasonable for the physician role. Second, the study advances illegitimate task research by positioning unreasonable tasks as a mechanism linking underused capability to moral distress rather than treating illegitimate tasks only as independent stressors or outcomes of poor work design. Third, the study extends moral distress research by linking moral distress to job crafting. This places moral distress is not only a well-being problem but also a self-regulatory turning point that is associated with lower growth-oriented crafting and higher protective crafting. Fourth, the study refines boundary-condition logic by showing why identity can operate in two different ways. Calling may heighten distress sensitivity when role-incongruent tasks obstruct meaningful professional work, while professional identity may help physicians maintain role-consistent action under moral strain.

## 2 Theoretical framework

### 2.1 Conservation of resources theory as the backbone

Conservation of resources theory explains stress as a function of threatened or actual loss of valued resources, and it predicts that people act to conserve what they have and regain what they lose (Hobfoll, 2001; Hobfoll, 1989). This lens aligns with the study’s model because each stage of the proposed process can be framed as resource dynamics. Perceived overqualification can be understood as a condition in which essential resources, such as expertise, mastery, and capability, exist but are blocked from yielding returns, such as recognition, autonomy, and meaningful contribution (Erdogan and Bauer, 2009; Woo, 2020). When blocked resources cannot be converted into valued outcomes, individuals experience strain and become more sensitive to additional losses.

Unreasonable illegitimate tasks can intensify this process because they consume scarce resources, such as time, energy, and attention, and they can also threaten social resources, such as respect and status. These tasks signal that the organization does not appropriately value the professional role, which is both a relational loss and a symbolic loss (Semmer et al., 2019; Semmer et al., 2015). COR theory also anticipates that when resource loss accumulates, people often shift toward defensive coping, disengagement, and withdrawal-oriented strategies (Hobfoll, 2001). In job design terms, this aligns with a stronger pull toward avoidance crafting and a weaker capacity for sustained approach crafting when distress becomes chronic or intense (Lin et al., 2023).

### 2.2 Job crafting theory as the behavioral response framework

Job crafting theory explains how employees proactively alter aspects of their jobs to improve person–job fit and sustain functioning (Wrzesniewski and Dutton, 2001). Classic job crafting describes changes to tasks, relationships, and the cognitive framing of work that employees use to increase meaning, resources, and control (Tims et al., 2012; Wrzesniewski and Dutton, 2001). In clinical contexts, job crafting can include redesigning routines, renegotiating responsibilities, seeking feedback or collaboration, and reframing patient care tasks to sustain professional meaning (Lin et al., 2023; Tims et al., 2012).

Job crafting distinguishes between approach and avoidance crafting. Approach crafting reflects proactive expansion of resources and challenges that support growth and motivation, and avoidance crafting reflects efforts to reduce hindering demands or emotionally depleting elements of work (Lin et al., 2023; Petrou et al., 2012). This distinction is essential to this study’s framework because POQ can increase motivation to craft, whereas moral distress can push crafting toward self-protection rather than growth-oriented redesign.

### 2.3 Stress-as-offense-to-self as the meaning mechanism behind illegitimate tasks

Stress-as-offense-to-self clarifies why illegitimate tasks are especially powerful. The core idea is that some stressors harm people because they convey disrespect and violate expectations about what someone in a given role should do. Illegitimate tasks are therefore not only extra work. They are experienced as an affront to the self, which is particularly salient for professionals whose work identity is tied to competence, service, and ethical standards (Semmer et al., 2019). This aligns closely with the illegitimate tasks framing in the literature, which emphasizes that illegitimate tasks violate role boundaries and can infringe on professional identity (Liu et al., 2024; Ma and Xie, 2024).

For doctors, unreasonable illegitimate tasks are likely to be interpreted as organizational disregard for professional time and expertise, which increases emotional conflict and fuels moral distress (Morley et al., 2019).

### 2.4 Identity-based theories and why calling and professional identity matter

Identity-based theories explain how people interpret experiences through the lens of who they are and what their role means (Ashforth and Mael, 1989; Pratt et al., 2006). Professional identity is a salient aspect of doctors’ identity, and it shapes how they evaluate tasks, ethical constraints, and acceptable coping strategies (Cruess et al., 2014). When professional identity is strong, people often protect identity-congruent behavior and resist acting in ways that feel inconsistent with the role.

Calling can operate similarly, but in a dual way that fits the specific hypothesis. Calling can function as a motivational resource that sustains meaning (Dik and Duffy, 2009; Dik et al., 2012). At the same time, calling can raise standards and moral sensitivity, so constraints and illegitimate demands can feel more offensive and more distressing (Dobrow and Tosti-Kharas, 2011). Studies align calling orientation with COR-based resource processes and differential effects on approach versus avoidance job crafting (Jiang D. K. et al., 2024; Kim, 2025).

Thus, these perspectives explain why the proposed model is serial and moderated rather than a direct or single mediator. The constructs are related, but they are not interchangeable. Perceived overqualification captures a person-job misfit appraisal. Unreasonable, illegitimate tasks capture a role-legitimacy appraisal. Moral distress captures the ethical strain linked to constrained professional standards. Approach and avoidance job crafting capture behavioral self-regulation. This sequencing matters because what physicians are asked to do is conceptually different from how those demands are ethically experienced, and both are distinct from how physicians adjust their work afterward. A direct perceived overqualification to the job crafting model would show whether overqualified physicians craft their jobs, but it would not explain how underused expertise becomes ethically consequential through role-incongruent task assignments. A single-mediator model would collapse role-violation appraisal and moral distress, although the former concerns assigned work and the latter concerns ethical strain. A model without calling and professional identity would miss why the same task condition may be more distressing for highly called physicians and less likely to redirect behavior among physicians with a stronger professional identity. The model, therefore, tests a clinical role-legitimacy process rather than a generic stress-response chain.

### 2.5 Hypotheses development

#### 2.5.1 Perceived overqualification and approach job crafting and avoidance job crafting

Doctors who feel overqualified often perceive a mismatch between their capabilities and the opportunities their roles provide to apply those capabilities. Job crafting theory suggests that employees respond to such misfit by proactively redesigning aspects of their work to increase challenge, control, and meaning (Wrzesniewski and Dutton, 2001). From a conservation-of-resources perspective, approach crafting can be understood as an investment strategy that converts underutilized human capital into valued resources, such as autonomy, effectiveness, and recognition (Hobfoll, 2001). Empirical studies indicate that perceived overqualification can be associated with more proactive job redesign behaviors, particularly when employees attempt to restore fit and reclaim meaningful use of their skills (Debus et al., 2020; Kim et al., 2020; Şeşen and Ertan, 2020).

Additionally, although overqualification can motivate constructive redesign, it can also create strain when surplus capability cannot be translated into valued outcomes and when the environment constrains discretion. COR theory predicts that when resource gain is blocked and loss pressure increases, individuals may shift toward protective regulation that limits further depletion (Hobfoll, 2001). In job crafting terms, this implies stronger tendencies to reduce exposure to hindering demands, emotionally draining interactions, or tasks perceived as low value, which aligns with avoidance crafting (Petrou et al., 2012; Seppälä et al., 2020). Prior evidence shows that perceived overqualification is linked to withdrawal-oriented and defensive patterns under constrained or unfair work conditions, suggesting that crafting may include demand-reduction responses rather than purely growth-oriented redesign (Debus et al., 2020; Fan and Shang, 2025). Therefore, I hypothesize that:

*H1*. Perceived overqualification is positively related to approach job crafting.

*H2*. Perceived overqualification is positively related to avoidance job crafting.

#### 2.5.2 Perceived overqualification and unreasonable illegitimate tasks

Stress-as-Offense-to-Self theory argues that some demands are stressful not only because they require effort, but because they imply disrespect and violate role-based expectations about what one should reasonably be asked to do (Semmer et al., 2015). Overqualified employees are likely to be particularly sensitive to such signals because misallocating their skills makes role boundary violations more salient and more likely to be interpreted as illegitimate. Empirical work shows that perceived overqualification is positively associated with perceptions of illegitimate tasks, reflecting a heightened appraisal that assigned duties are unreasonable relative to one’s role and competence (Fan et al., 2023). In medicine, where professional standards and specialized expertise strongly define role boundaries, feeling overqualified should therefore increase the likelihood that burdensome non-core requests are interpreted as unreasonable, illegitimate tasks. Hence, I argue that:

*H3*. Perceived overqualification is positively associated with perceived unreasonable, illegitimate tasks.

#### 2.5.3 Unreasonable illegitimate tasks and moral distress

Moral distress arises when clinicians recognize what they believe is ethically appropriate but are constrained by organizational conditions, competing demands, or systemic barriers (Epstein and Hamric, 2009; Morley et al., 2019). This construct is distinct from unreasonable, illegitimate tasks. Unreasonable, illegitimate tasks describe the physician’s appraisal of an assigned task as role-incongruent or inappropriate. Moral distress describes the ethical strain that may follow when such demands interfere with professional standards, clinical responsibility, or appropriate patient care. In clinical contexts, unreasonable tasks can divert time, energy, and attention away from core professional obligations, thereby increasing the perceived conflict between what physicians believe they should do and what their work conditions allow them to do. Evidence further suggests that illegitimate tasks shape appraisal and strain responses, which supports their role as a proximal work condition associated with moral distress when demands are perceived as improper (Ma and Xie, 2024).

*H4*. Perceived unreasonable, illegitimate tasks are positively related to moral distress.

#### 2.5.4 Moral distress and approach job crafting and avoidance job crafting

Job crafting requires self-regulatory capacity, perceived control, and an expectation that effortful redesign will produce meaningful gains (Wrzesniewski and Dutton, 2001). Moral distress is a psychologically depleting state characterized by conflict, constrained action, and repeated experiences of compromised standards, which COR theory conceptualizes as resource-loss pressure that undermines proactive investment. Empirically, moral distress is associated with burnout-related outcomes and impaired occupational functioning, indicating reduced capacity for sustained constructive engagement (Epstein et al., 2019; Morley et al., 2019). In healthcare samples, job crafting is generally beneficial for engagement and retention, but its enactment depends on available resources and supportive conditions (Rodríguez-García et al., 2024; Topa and Aranda-Carmena, 2022). Accordingly, as moral distress intensifies, doctors should be less able and less willing to engage in approach crafting that requires additional cognitive and emotional investment. Moral distress is also distinct from avoidance job crafting. Moral distress is a psychological state of ethical strain, whereas avoidance job crafting is an enacted work-redesign behavior. The former concerns how physicians experience constrained professional standards, while the latter concerns what physicians do to reduce hindering or depleting job demands. This distinction matters because moral distress does not automatically imply avoidance. Some physicians may respond to moral distress by seeking resources, reframing work, or maintaining role-consistent engagement, particularly when professional identity is strong. This model therefore treats avoidance crafting as one possible behavioral response to moral distress, not as another label for the same experience.

Moreover, when distress escalates and perceived control is limited, COR theory predicts a shift toward defensive coping that reduces further depletion. Avoidance job crafting, which emphasizes demand reduction and exposure minimization, is consistent with this protective pattern, particularly under sustained hindrance conditions (Seppälä et al., 2020). Moral distress is often linked to emotional exhaustion and withdrawal-oriented tendencies, suggesting that doctors may manage functioning by reducing engagement with draining or non-essential demands rather than expanding challenges (Epstein et al., 2019). Thus, moral distress should increase avoidance crafting aimed at conserving remaining resources in the face of continued ethical and workload pressures. Therefore, I hypothesize that:

*H5*. Moral distress is negatively associated with approach job crafting.

*H6*. Moral distress is positively associated with avoidance job crafting.

#### 2.5.5 The mediating role of perceived unreasonable illegitimate tasks and moral distress

The proposed serial pathway integrates resource loss and meaning-based threat mechanisms. Perceived overqualification increases sensitivity to role misallocation and boundary violations, thereby elevating perceptions of unreasonable, illegitimate tasks (Fan et al., 2023). These tasks then operate as disrespecting, resource-consuming stressors that intensify conflict between professional ideals and constrained practice, increasing moral distress (Morley et al., 2019). As distress accumulates, doctors should prioritize conservation, which, behaviorally, translates into job crafting that reduces exposure to further depletion (Seppälä et al., 2020). This logic is consistent with evidence that illegitimate tasks shape appraisals and reactions in ways that promote strain, and that overqualification can initiate adverse downstream processes when work conditions block resource gain.

Thus, the same upstream process should undermine growth-oriented redesign. POQ increases the likelihood that doctors perceive role-incongruent duties as unreasonable, illegitimate tasks, reinforcing a sense of disrespect and misrecognition. These appraisals heighten moral distress by constraining agency and conflicting with professional standards. Under resource loss pressure, individuals are less likely to invest in approach-oriented crafting that requires discretionary effort and optimistic expectations of gain. Empirical evidence that moral distress co-occurs with burnout-related outcomes and impaired functioning further supports a reduced capacity for proactive, expansive forms of job redesign (Epstein et al., 2019). Therefore, I hypothesize that:

*H7*. POQ has a positive indirect effect on avoidance job crafting via perceived unreasonable, illegitimate tasks and moral distress.

*H8*. POQ has a negative indirect effect on approach job crafting via perceived unreasonable, illegitimate tasks and moral distress.

#### 2.5.6 Calling as a moderator

Calling reflects a strong sense that one’s work is meaningful, identity-relevant, and morally significant (Dik et al., 2012). Calling can operate as a motivational resource, but it can also intensify strain when work conditions prevent individuals from enacting their values because the stakes of role interference are higher. This “double-edged” logic is consistent with research that frames calling as beneficial in supportive contexts but potentially costly under constrained or stressful conditions where values cannot be enacted (Dobrow and Tosti-Kharas, 2011). For doctors with a high calling, unreasonable, illegitimate tasks should be especially offensive because they divert time and moral attention from patient care and undermine the enactment of professional purpose. This should heighten the distress response to illegitimate tasks. Therefore, I hypothesize that:

*H9*. Calling strengthens the positive association between perceived unreasonable, illegitimate tasks and moral distress, such that this association is stronger for doctors with higher calling than for those with lower calling.

#### 2.5.7 Professional identity as a moderator for approach job crafting and avoidance job crafting

Professional identity is a salient self-definition that guides how clinicians interpret demands, regulate behavior, and protect role-consistent standards (Ashforth and Mael, 1989; Cruess et al., 2014). When professional identity is strong, doctors may be more likely to maintain constructive engagement and uphold identity-consistent actions even under moral strain because disengagement would itself threaten the self-concept associated with being a competent and ethical physician. This implies that professional identity can function as a buffering resource that sustains purposeful action under distress, reducing the extent to which moral distress suppresses approach crafting. Evidence in healthcare contexts also emphasizes the importance of professional identity for resilience and adaptive responses under ethically challenging conditions.

Furthermore, professional identity should also shape whether distress translates into a protective withdrawal-oriented redesign. Since avoidance crafting can involve reducing engagement with aspects of work that are emotionally or cognitively demanding, it may be interpreted by highly identified doctors as inconsistent with professional standards of responsibility and service. Identity-based perspectives predict that strong identification motivates behavior that preserves role integrity and discourages coping patterns that imply disengagement from core role obligations (Pratt et al., 2006). Thus, even when moral distress is elevated, doctors with a stronger professional identity should be less likely to respond by increasing avoidance crafting and more likely to remain anchored to role-consistent engagement strategies. Therefore, I hypothesize that:

*H10*. Professional identity attenuates the negative relationship between moral distress and approach job crafting, making this association weaker for doctors with high professional identity than for those with low professional identity.

*H11*. Professional identity attenuates the positive relationship between moral distress and avoidance job crafting, making this association weaker for doctors with high professional identity than for those with low professional identity.

**Figure 1:**
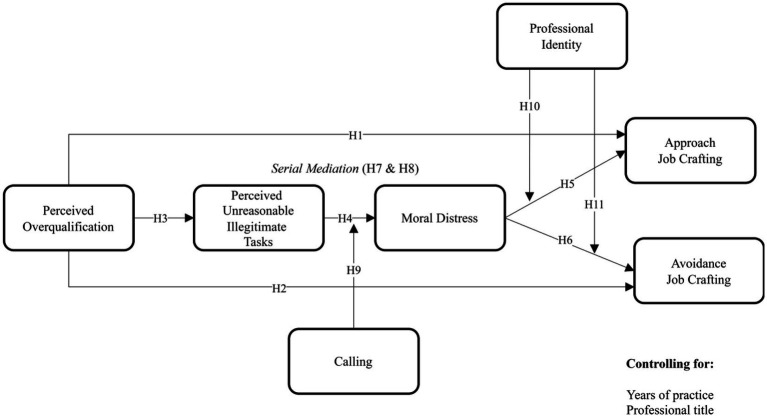
Conceptual framework.

Figure 1 shows the conceptual framework of the study.

## 3 Materials and methods

### 3.1 Sample and procedure

This study used a cross-sectional observational design. Practicing physicians were recruited from several top-tier tertiary hospitals in Sichuan Province, Chengdu, to test the proposed mechanisms. Tertiary hospitals occupy the highest level in China’s hospital classification system and represent demanding clinical settings in which physicians typically have advanced training, specialized expertise, and high professional responsibility. This sampling frame was appropriate for the present study because the model focuses on physicians’ perceived overqualification, role-incongruent task assignment, moral distress, and job crafting under high professional demands. I used hospital-based, clustered recruitment, in which formal gatekeepers within each participating hospital, such as department heads, medical affairs offices, or administrators, disseminated a secure survey link to eligible doctors. Data were collected through an anonymous, self-administered web questionnaire that presented an information sheet and required electronic consent before participants accessed the items. In line with established reporting guidance for online surveys, I applied quality protections, such as restricting submissions to one response per device or link, excluding implausibly fast completions, and using attention checks. A total of 458 responses were received, and 437 were retained for analysis after applying pre-specified eligibility and data-quality screening. Since single-source, single-time surveys are prone to common method variance, the questionnaire emphasized confidentiality, used neutral item wording, and psychologically separated the predictor and outcome sections to reduce evaluation apprehension and response consistency artifacts (Eysenbach, 2004; Podsakoff et al., 2003; Von Elm et al., 2007).

### 3.2 Sample size adequacy

A RMSEA close-fit power check indicated that the sample easily supports both the CFA and the structural SEM. With *N* = 437, power was 1.00 for both models under RMSEA contrasts of 0.05 vs. 0.08 and 0 vs. 0.05. The N needed for 0.80 power was 29.4–42.5 for the CFA and 21.6–31.7 for the structural model. This follows Maccallum et al.’s (1996) RMSEA-based non-central chi-square approach, where power depends on N and df. The cases-per-indicator ratio was 8.4:1, which falls within the common 5–10 guideline and supports adequate indicator coverage for the CFA.

### 3.3 Measures

#### 3.3.1 Perceived overqualification

Perceived overqualification was measured using the nine-item scale developed by Maynard et al. (2006). The scale captures employees’ subjective perceptions that their education, skills, abilities, and work experience exceed the requirements of their current job. Sample items include statements such as “My educational level is higher than what my job requires” and “Much of my knowledge is not used in my current position.” Respondents indicated their level of agreement with each statement on a 5-point Likert scale ranging from 1 (strongly disagree) to 5 (strongly agree). Higher scores reflect stronger perceptions of overqualification.

#### 3.3.2 Job crafting

I measured job crafting with the 21-item Job Crafting Scale (Tims et al., 2012). The scale captures self-initiated changes to job demands and resources across four behaviors: increasing structural resources (five items), increasing social resources (five items), increasing challenging demands (five items), and decreasing hindering demands (six items). Respondents rated each item from 1 (never) to 5 (often). For the structural model, I formed two outcomes: approach job crafting, combining the three “increase” dimensions, and avoidance job crafting, using the “decrease hindering demands” dimension.

#### 3.3.3 Unreasonable illegitimate tasks

Unreasonable illegitimate tasks were assessed with the unreasonable tasks subscale of the Bern Illegitimate Tasks Scale using four items that capture tasks perceived as unnecessary or non-sensical (e.g., whether tasks “have to be done at all”) rated on a 5-point frequency scale from 1 (never) to 5 (often). Item scores were averaged, with higher values indicating more unreasonable, illegitimate tasks (Semmer et al., 2015).

#### 3.3.4 Moral distress

Moral distress was measured with nine items from the Measure of Moral Distress for Healthcare Professionals (MMD-HP). Each situation was rated for frequency, from 0 (never) to 4 (frequently), and for distress intensity, from 0 (none) to 4 (distressing). Consistent with the instrument’s scoring logic, item-level moral distress impact was computed as the product of frequency and distress, with higher scores indicating greater moral distress (Epstein et al., 2019).

#### 3.3.5 Professional identity

Professional identity was measured using the seven-item Professional Self Identity Questionnaire, rated on a 5-point agreement scale from 1 (strongly disagree) to 5 (strongly agree). Scores were averaged such that higher values indicated stronger professional identity (Crossley and Vivekananda-Schmidt, 2009).

#### 3.3.6 Calling (presence)

Calling was measured with the two presence items from the Brief Calling Scale (Vaske et al., 2017). This measure was selected because the study focuses on the presence of calling as a boundary condition rather than the search for calling or the broader multidimensional structure of calling. Participants responded on a 5-point scale from 1, not at all true of me, to 5, totally true of me. Higher scores indicated a stronger sense of calling. For two-item measures, reliability is more appropriately assessed through the inter-item correlation and the Spearman–Brown coefficient than through Cronbach’s alpha alone (Vaske et al., 2017). The two items correlated at *r* = 0.57, and the Spearman-Brown reliability was acceptable, ρSB = 0.73. These results supported the use of the composite in the CFA, SEM, and latent interaction models, while recognizing that the measure captures the presence dimension of calling only.

#### 3.3.7 Controls

I controlled for years in practice and professional title because experience and hierarchy are associated with ethical strain and moral distress in clinical work. They can shape how clinicians respond to constraints and authority structures (Dodek et al., 2016). Additionally, job crafting differs across career stages and professional titles in healthcare, so adjusting for these factors reduces confounding in the paths to approach and avoidance crafting (Wang et al., 2025).

### 3.4 Data analysis

I estimated all models in R using a fully script-based workflow to ensure transparency and reproducibility (R Core Team, 2000). I fit the SEMs in lavaan (Rosseel, 2012). Since Likert indicators and product terms often deviate from normality, I estimated the primary model with robust maximum likelihood (MLR) to obtain robust standard errors and a scaled test statistic (Satorra and Bentler, 1994; Yuan and Bentler, 2000). I then refit the same model using ML and 10,000 non-parametric bootstrap resamples and report bias-corrected confidence intervals for indirect and conditional indirect effects (Efron, 1987; Preacher et al., 2007). I specified latent interactions with the product–indicator approach using *semTools indProd* and applied double mean-centering to reduce non-essential multicollinearity (Kenny and Judd, 1984; Lin et al., 2010). I used all feasible pairwise products for the first interaction and matched products for the second to limit indicator growth without changing the moderation test (Marsh et al., 2004). I tested moderated serial mediation using model-defined parameters, such as conditional indirect effects at −1 SD, the mean, and +1 SD of the moderators, and I based inference on robust estimates and bootstrap intervals (Hayes, 2015; Preacher et al., 2007). Years of Practice and professional title/rank entered as covariates on all endogenous latent outcomes.

## 4 Results

The sample was concentrated among male and senior physicians. Specifically, 408 respondents were male (93.36%), 302 were associate chief or chief physicians (69.11%), and 356 had at least 11 years of practice (81.46%). This composition indicates that the analytic sample primarily reflects experienced physicians in top-tier tertiary hospital settings. The demographic distribution of the sample is shown in Table 1.

**Table 1:** Respondents demographics.

Variable	Category	*n*	%
Gender	Female	29	6.64
	Male	408	93.36
Age (years)	21–30	27	6.18
	31–40	128	29.29
	41–50	224	51.26
	51–60	58	13.27
Education	Bachelor’s degree	235	53.77
	Master’s degree	166	37.98
	Doctoral degree	36	8.24
Years of practice	<1 year	2	0.46
	1–5 years	38	8.7
	6–10 years	41	9.38
	11–20 years	168	38.44
	≥20 years	188	43.02
Professional title	Physician	43	9.84
	Attending physician	92	21.05
	Associate chief/Chief physician	302	69.11

### 4.1 Measurement model assessment

A maximum-likelihood EFA with oblimin rotation supported a seven-factor structure. Items loaded strongly on their intended factors (*λ* ≈ 0.63–0.88) with negligible cross-loadings, indicating clear factors (Costello and Osborne, 2005). All seven factors had eigenvalues above 1 (12.08 to 1.41) and explained 65.1% of the variance (Fabrigar et al., 1999). A seven-factor CFA using MLR (*N* = 437) showed excellent fit: robust χ^2^ (1,253) = 1,418.13, *p* = 0.001, CFI = 0.990, TLI = 0.990, RMSEA = 0.018, 90% CI [0.012, 0.022], SRMR = 0.031 (Hu and Bentler, 1999).

#### 4.1.1 Reliability and validity

All constructs showed adequate reliability and convergent validity. Cronbach’s *α* and composite reliability ranged from 0.73 to 0.97, and AVE ranged from 0.54 to 0.73, all above recommended cutoffs (Fornell and Larcker, 1981; Hair et al., 2009). HTMT supported discriminant validity: all ratios were below 0.85 (range = 0.036–0.456; max = 0.456 for POQ–UIT), and 2,000-sample BCa bootstrap intervals did not include 0.85 for any pair (Henseler et al., 2015). This supports the empirical distinctiveness of the seven constructs, such as the theoretically close constructs of unreasonable illegitimate tasks, moral distress, and avoidance job crafting (see Table 2). The results, therefore, reduce the concern that the model is capturing a single undifferentiated negative work experience rather than separable task appraisal, ethical strain, and behavioral response constructs.

**Table 2:** Measurement model assessment.

Construct	Indicator	Loading (Std.)	Indicator *R*^2^	CA (α)	CR	AVE
POQ	POQ1	0.841	0.707	0.955	0.955	0.704
	POQ2	0.847	0.718			
	POQ3	0.826	0.683			
	POQ4	0.845	0.714			
	POQ5	0.847	0.716			
	POQ6	0.834	0.695			
	POQ7	0.843	0.711			
	POQ8	0.832	0.692			
	POQ9	0.835	0.697			
APJC	APJC1	0.843	0.708	0.968	0.969	0.673
	APJC2	0.807	0.648			
	APJC3	0.84	0.704			
	APJC4	0.808	0.65			
	APJC5	0.803	0.641			
	APJC6	0.82	0.671			
	APJC7	0.823	0.675			
	APJC8	0.831	0.687			
	APJC9	0.822	0.672			
	APJC10	0.828	0.683			
	APJC11	0.796	0.631			
	APJC12	0.815	0.662			
	APJC13	0.803	0.641			
	APJC14	0.838	0.698			
	APJC15	0.824	0.676			
AVJC	AVJC1	0.751	0.563	0.901	0.902	0.606
	AVJC2	0.789	0.621			
	AVJC3	0.795	0.629			
	AVJC4	0.758	0.574			
	AVJC5	0.789	0.621			
	AVJC6	0.785	0.613			
UIT	UIT1	0.764	0.586	0.856	0.857	0.6
	UIT2	0.781	0.607			
	UIT3	0.799	0.636			
	UIT4	0.754	0.569			
PI	PI1	0.7	0.489	0.889	0.89	0.538
	PI2	0.732	0.537			
	PI3	0.737	0.544			
	PI4	0.77	0.591			
	PI5	0.813	0.659			
	PI6	0.692	0.479			
	PI7	0.672	0.452			
MD	MD1_	0.817	0.667	0.959	0.96	0.726
	MD2_	0.811	0.657			
	MD3_	0.857	0.734			
	MD4_	0.846	0.714			
	MD5_	0.898	0.806			
	MD6_	0.859	0.738			
	MD7_	0.849	0.719			
	MD8_	0.869	0.753			
	MD9_	0.841	0.707			
CALL	BCS1	0.769	0.582	0.726	0.726	0.57
	BCS2	0.741	0.558			

UIT, Unreasonable illegitimate tasks; APJC, Approach job crafting; AVJC, Avoidance job crafting; POQ, Perceived overqualification; MD, Moral distress; PI, Professional identity; CALL, Calling.

### 4.2 Descriptive statistics and correlations

Item means were near the scale midpoint (*M* = 2.90–3.44). POQ, APJC, AVJC, UIT, PI, and CALL items were approximately symmetric (skew = −0.23 to 0.22; kurtosis = −0.77 to 0.32). Moral distress (MD) indicators were strongly right-skewed and leptokurtic (skew = 1.94–2.60; kurtosis = 3.41–7.97) because each item is an impact score computed as frequency × distress (0–16). The same pattern held for scale composites: POQ, APJC, AVJC, UIT, PI, and CALL were well-behaved (*M* = 2.96–3.01; SD = 0.61–0.97; |skew| ≤ 0.12; kurtosis = −0.64 to 0.21), whereas MD remained non-normal (*M* = 3.13, SD = 2.84, skew = 2.35, kurtosis = 5.82). I therefore used MLR to obtain robust standard errors and a scaled test statistic under non-normality (Rosseel, 2012).

**Figure 2:**
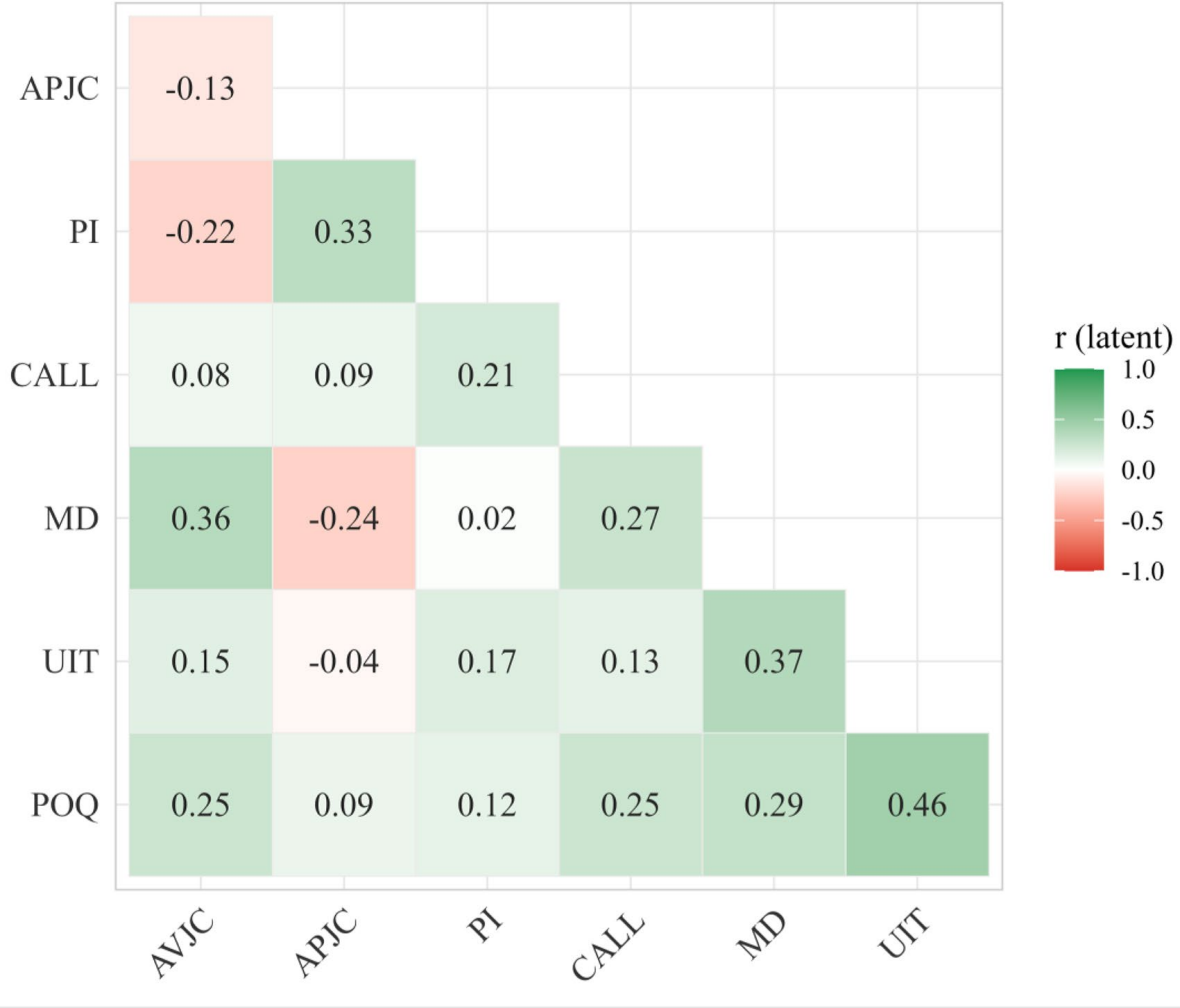
Correlation matrix heatmap.

Factor correlations were significant but modest (|*r*| = 0.02–0.46), supporting empirical distinctiveness rather than construct redundancy. This is especially relevant for the theoretically close constructs since POQ–UIT (*r* = 0.46) and UIT–MD (*r* = 0.37) were related but not so highly correlated as to suggest empirical overlap (Kline, 2023) (see Figure 2).

### 4.3 Common method variance

Harman’s single-factor test indicated that the first factor accounted for 21.62% of the total variance (SS loadings = 11.24), which is below the standard 50% heuristic for problematic common method variance. Consistent with this, the unmeasured latent method factor (CLF/ULMC) model fit improved only slightly relative to the baseline CFA (CFI = 0.992 vs. 0.989; TLI = 0.991 vs. 0.988; RMSEA = 0.016 vs. 0.018; SRMR = 0.028 vs. 0.031), suggesting that common method bias is unlikely to be materially driving the results, while recognizing that these *post hoc* diagnostics have known limitations (Podsakoff et al., 2012).

### 4.4 Hypothesis testing

I estimated the structural model with robust maximum likelihood (MLR). The model fit the data well: χ^2^ (2,246) = 3,532.24, *p* < 0.001, robust CFI = 0.94, robust TLI = 0.93, robust RMSEA = 0.04 (90% CI [0.04, 0.04]), and SRMR = 0.04, meeting standard cutoffs. The model accounted for meaningful variance in the endogenous constructs (R^2^ = 0.20–0.24: UIT = 0.21, MD = 0.24, APJC = 0.20, AVJC = 0.21), which is typical for an organizational model (Kline, 2023).

#### 4.4.1 Direct effects

**Figure 3:**
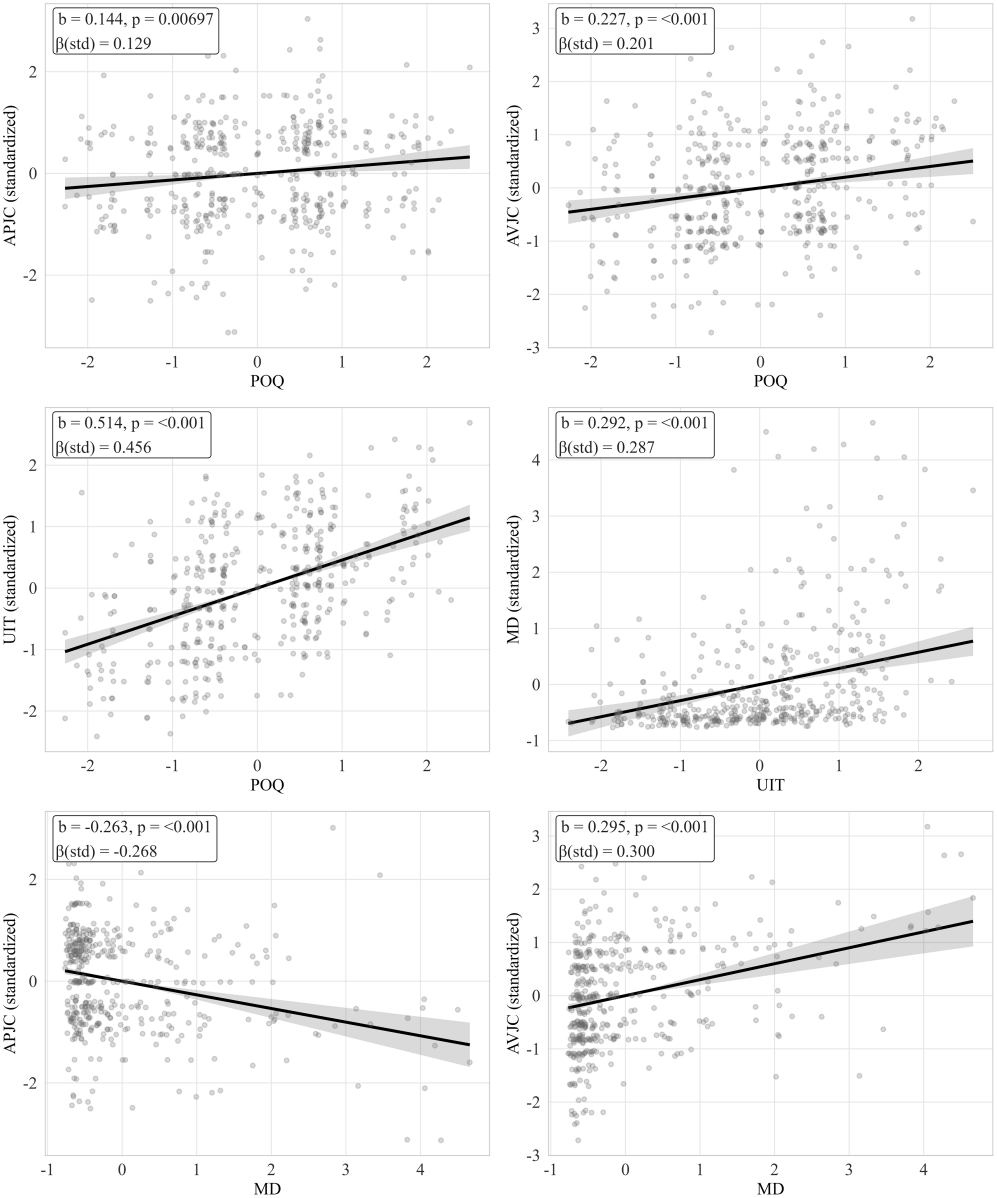
Direct paths slopes.

POQ was positively associated with UIT (*β* = 0.456, *p* < 0.001), supporting H3. POQ was also positively associated with APJC (*β* = 0.129, *p* = 0.007), supporting H1, and with AVJC (*β* = 0.201, *p* < 0.001), supporting H2. UIT was positively associated with MD (*β* = 0.287, *p* < 0.001), supporting H4. MD was negatively associated with APJC (*β* = −0.268, *p* < 0.001), supporting H5, and positively associated with AVJC (*β* = 0.300, *p* < 0.001), supporting H6 (see Table 3 and Figure 3).

**Table 3:** Path coefficients.

Predictor	*b*	SE	*z*	*p*	Std. *β*	95% BCa CI (b)	*f* ^2^
Direct							
POQ → UIT	0.514***	0.062	8.3	<0.001	0.456	[0.391, 0.638]	0.262
UIT → MD	0.292***	0.056	5.22	<0.001	0.287	[0.180, 0.403]	0.075
POQ → APJC	0.144**	0.053	2.7	0.007	0.129	[0.036, 0.248]	0.017
MD → APJC	−0.263***	0.049	−5.33	<0.001	−0.268	[−0.363, −0.169]	0.069
POQ → AVJC	0.227***	0.058	3.9	<0.001	0.201	[0.109, 0.342]	0.046
MD → AVJC	0.295***	0.056	5.32	<0.001	0.3	[0.186, 0.411]	0.09
Interaction							
UIT × CALL → MD	0.252***	0.05	5.01	<0.001	0.22	[0.148, 0.360]	0.059
MD × PI → APJC	0.163*	0.064	2.53	0.011	0.146	[0.043, 0.300]	0.018
MD × PI → AVJC	−0.104†	0.057	−1.84	0.067	−0.093	[−0.223, 0.014]	0.006

Years of practice and professional title or rank were entered as covariates on all endogenous latent outcomes. †*p* < 0.10. *p < 0.05. **p < 0.01. ***p < 0.001.

#### 4.4.2 Moderation effects

**Figure 4:**
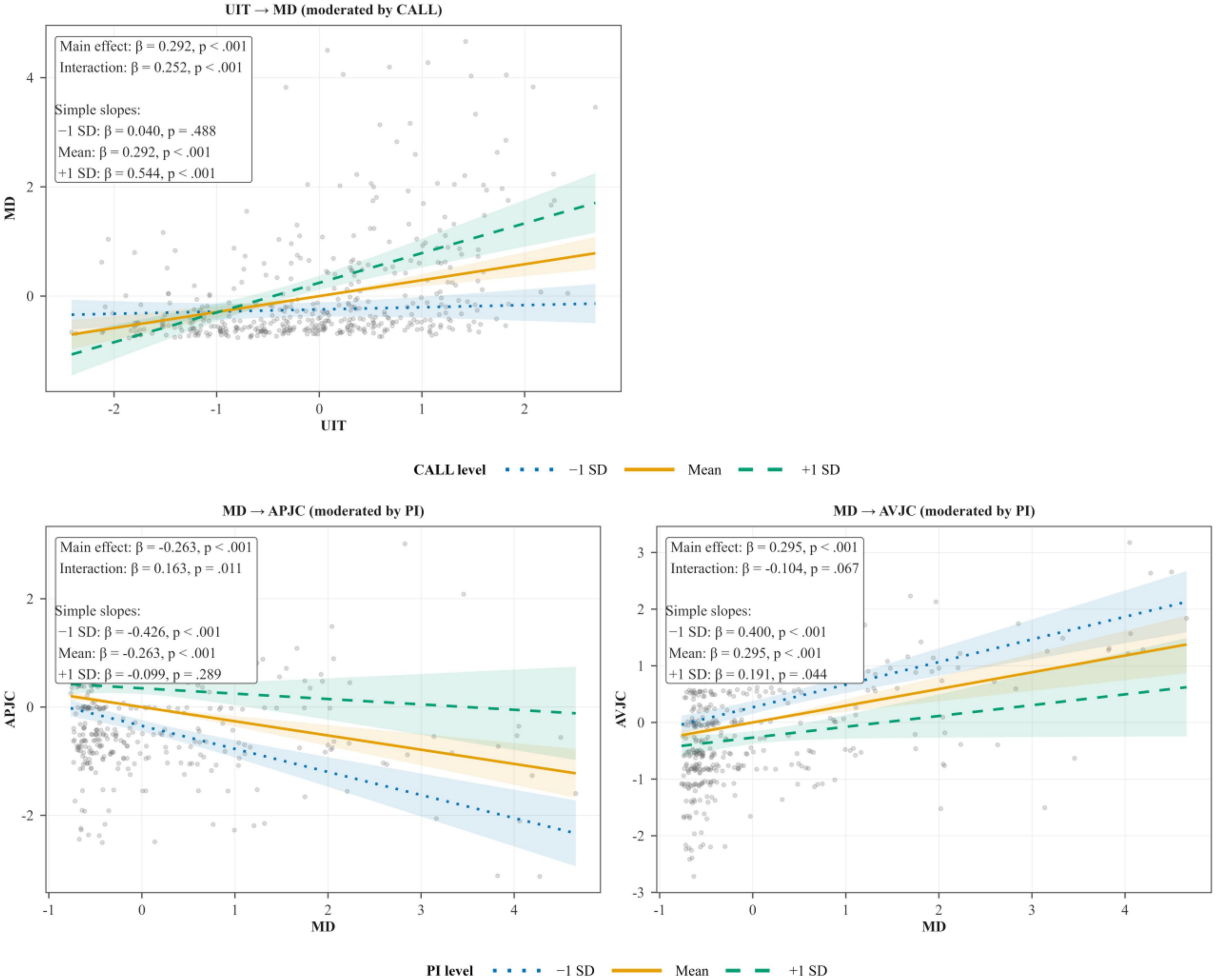
Moderation simple slopes.

The UIT × CALL interaction was positively associated with MD (*β* = 0.220, *p* < 0.001), supporting H9. The MD × PI interaction was positively associated with APJC (*β* = 0.146, *p* = 0.011), supporting H10. The MD × PI interaction was not statistically significant for AVJC at *p* < 0.05 (*β* = −0.093, *p* = 0.067), so H11 was not supported. Simple slopes showed that UIT was associated with MD at mean CALL (*β* = 0.292, *p* < 0.001) and high CALL (*β* = 0.544, *p* < 0.001), but not at low CALL (*β* = 0.040, *p* = 0.49). MD was associated with lower APJC at low PI (*β* = −0.426, *p* < 0.001) and mean PI (*β* = −0.263, *p* < 0.001), but not at high PI (*β* = −0.099, *p* = 0.29). MD was associated with higher AVJC at low PI (*β* = 0.400, *p* < 0.001), mean PI (*β* = 0.295, *p* < 0.001), and high PI (*β* = 0.191, *p* = 0.04) (see Figure 4).

#### 4.4.3 Mediation effects

For APJC, the serial indirect effect was significant and negative (*p* < 0.001, *β* = −0.035), supporting H8. In contrast, the total effect was not significant (*p* = 0.156, *β* = 0.066) based on the BCa bootstrap criterion for indirect effects.

For AVJC, the serial indirect effect was significant and positive (*p* < 0.001, *β* = 0.039), supporting H7, and the total effect was significant (*p* < 0.001, *β* = 0.272). Substantively, the APJC model reflects competitive (inconsistent) mediation because the direct effect is positive but the serial indirect effect is negative, whereas the AVJC model reflects complementary mediation because both the direct and serial indirect effects are positive (Zhao et al., 2010) (See Table 4).

**Table 4:** Mediation analysis.

Effect type	*b*	SE	*z*	*p*	Std. *β*	95% BCa CI (b)
Outcome: APJC						
Direct POQ → APJC	0.144**	0.053	2.7	0.007	0.129	[0.036, 0.248]
Indirect (serial) POQ → UIT → MD → APJC	−0.039***	0.01	−3.93	<0.001	−0.035	[−0.063, −0.022]
Total effect	0.074	0.052	1.42	0.156	0.066	[−0.030, 0.177]
Outcome: AVJC						
Direct POQ → AVJC	0.227***	0.058	3.9	<0.001	0.201	[0.109, 0.342]
Indirect (serial) POQ → UIT → MD → AVJC	0.044***	0.012	3.7	<0.001	0.039	[0.024, 0.073]
Total effect	0.306***	0.059	5.21	<0.001	0.272	[0.186, 0.422]

***p < 0.001, **p < 0.01, *p < 0.05, †*p* < 0.10. Years of practice and professional title/rank entered as covariates on all endogenous latent outcomes. UIT, unreasonable illegitimate tasks; APJC, approach job crafting; AVJC, avoidance job crafting; POQ, perceived overqualification; MD, moral distress; PI, professional identity; CALL, calling.

#### 4.4.4 Additional analysis (moderated serial mediation)

For APJC, moderated serial mediation was supported. The indices were significant for CALL at mean PI (*β* = −0.034, *p* < 0.001), PI at mean CALL (*β* = 0.025, *p* = 0.029), and dual moderation (*β* = 0.021, *p* = 0.046) (Hayes, 2018; Preacher et al., 2007).

**Figure 5:**
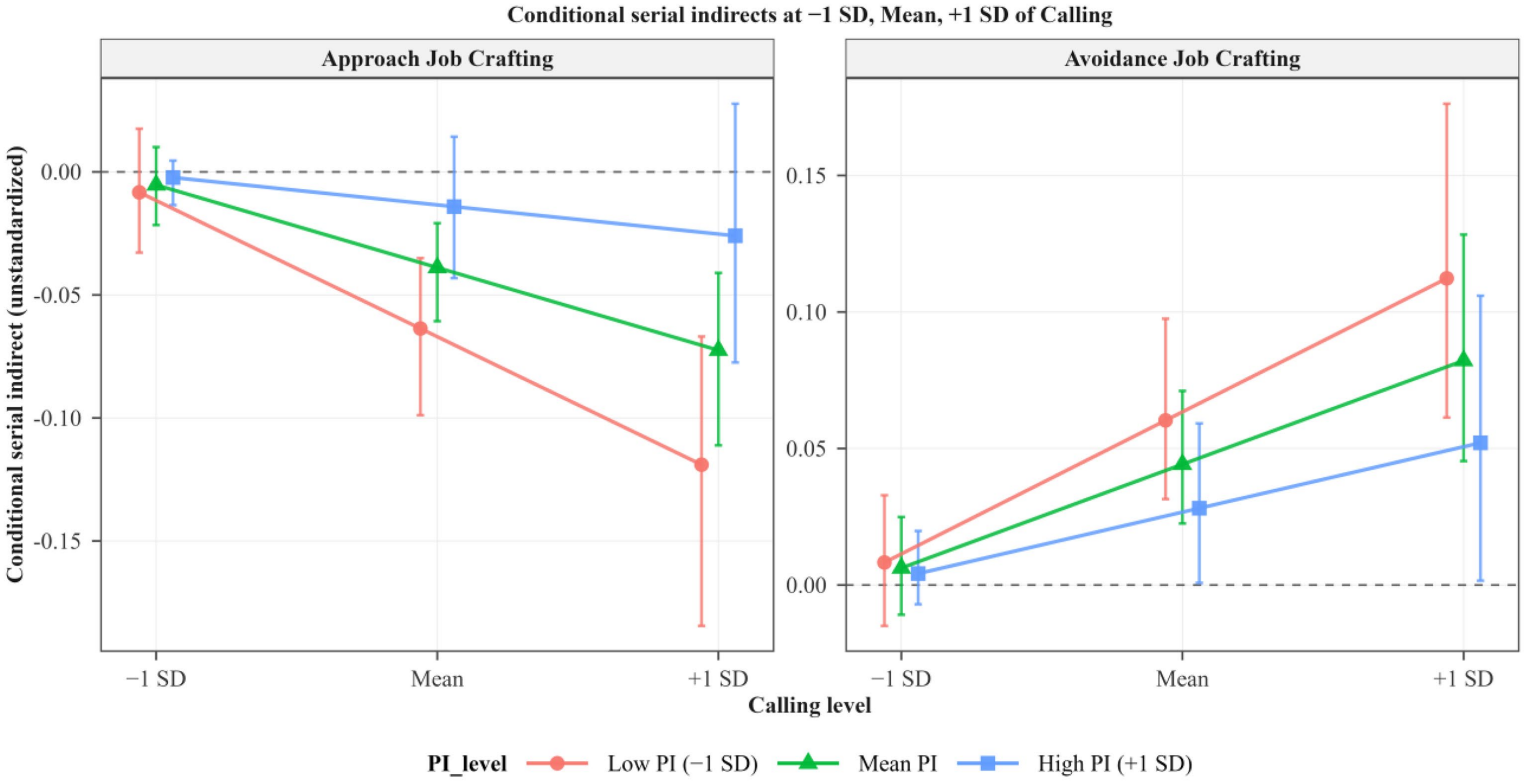
Conditional serial indirect effect plots.

For AVJC, moderated serial mediation was partially supported. The CALL at mean PI index was significant (*β* = 0.038, *p* < 0.001), but the PI at mean CALL (*β* = −0.016, *p* = 0.105) and dual moderation (*β* = −0.014, *p* = 0.121) indices were not significant (See Table 5). Figure 5 shows the same qualitative pattern. For APJC, the conditional serial indirect effect becomes more negative as CALL increases, and it is most negative under low PI while becoming closer to zero under high PI (with several high-PI intervals crossing zero). For AVJC, the conditional serial indirect effect becomes more positive as CALL increases, with the largest effects under low PI and smaller effects under high PI.

**Table 5:** Moderated serial mediation analysis.

Conditional indirect effects on approach job crafting (APJC)							
CALL level	PI level	*b*	SE	*z*	*p*	Std. *β*	95% BCa CI (b)
−1 SD	−1 SD	−0.009	0.012	−0.71	0.479	−0.013	[−0.036, 0.018]
−1 SD	Mean	−0.005	0.008	−0.69	0.488	−0.008	[−0.024, 0.010]
−1 SD	+1 SD	−0.002	0.004	−0.56	0.579	−0.004	[−0.019, 0.003]
Mean	−1 SD	−0.064***	0.016	−3.98	<0.001	−0.054	[−0.103, −0.036]
Mean	Mean	−0.039***	0.01	−3.93	<0.001	−0.035	[−0.063, −0.022]
Mean	+1 SD	−0.015	0.014	−1.07	0.286	−0.016	[−0.044, 0.014]
+1 SD	−1 SD	−0.119***	0.03	−4.02	<0.001	−0.096	[−0.193, −0.068]
+1 SD	Mean	−0.073***	0.018	−4.17	<0.001	−0.062	[−0.115, −0.042]
+1 SD	+1 SD	−0.028	0.025	−1.09	0.277	−0.028	[−0.079, 0.027]
CALL index at PI mean		−0.034***	0.009	−3.7	< 0.001	−0.027	[−0.057, −0.018]
PI index at CALL mean		0.025*	0.011	2.19	0.029	0.019	[0.006, 0.054]
Dual moderation index (CALL × PI)		0.021*	0.011	1.99	0.046	0.015	[0.005, 0.051]

Conditional indirect effects on avoidance job crafting (AVJC)							
−1 SD	−1 SD	0.008	0.012	0.69	0.488	0.012	[−0.017, 0.036]
−1 SD	Mean	0.006	0.009	0.68	0.495	0.009	[−0.011, 0.028]
−1 SD	+1 SD	0.004	0.006	0.64	0.523	0.006	[−0.006, 0.024]
Mean	−1 SD	0.060***	0.016	3.71	<0.001	0.051	[0.033, 0.099]
Mean	Mean	0.044***	0.012	3.7	<0.001	0.039	[0.024, 0.073]
Mean	+1 SD	0.029*	0.015	1.96	0.05	0.027	[0.002, 0.063]
+1 SD	−1 SD	0.112***	0.029	3.91	<0.001	0.091	[0.064, 0.182]
+1 SD	Mean	0.083***	0.021	4.03	<0.001	0.069	[0.047, 0.131]
+1 SD	+1 SD	0.053*	0.026	2.04	0.041	0.048	[0.002, 0.113]
CALL index at PI mean		0.038***	0.01	3.72	< 0.001	0.03	[0.020, 0.064]
PI index at CALL mean		−0.016	0.01	−1.62	0.105	−0.012	[−0.041, 0.001]
Dual moderation index (CALL × PI)		−0.014	0.009	−1.55	0.121	−0.009	[−0.037, 0.001]

***p < 0.001, **p < 0.01, *p < 0.05, †*p* < 0.10. Years of practice and professional title/rank entered as covariates on all endogenous latent outcomes. UIT, unreasonable illegitimate tasks; APJC, approach job crafting; AVJC, avoidance job crafting; POQ, perceived overqualification; MD, moral distress; PI, professional identity; CALL, calling.

#### 4.4.5 Robustness tests (alternative model comparison)

I estimated alternative models using MLR and compared fit (CFI, TLI, RMSEA, SRMR) and information criteria (AIC, BIC) (Rosseel, 2012; Schwarz, 1978). The hypothesized moderated serial mediation model provided the best overall balance of fit and parsimony among models estimated on the same indicator set. Adding direct paths from UIT to APJC and AVJC produced no meaningful improvement (ΔAIC = 2.37), while constraining direct effects or removing key stages of the mechanism worsened fit and increased AIC and BIC (Burnham and Anderson, 2002; Marsh et al., 2004; Symonds and Moussalli, 2011; Vrieze, 2012). A no-interactions model showed better apparent fit and lower AIC and BIC, but it is not directly comparable because it omits product–indicator variables and therefore changes the observed data structure; I treated it as a parsimonious baseline rather than a competing likelihood-based alternative.

These comparisons also need to be interpreted theoretically. A direct-effects model is less informative because it treats job crafting as an immediate response to perceived overqualification and omits the clinical route through role legitimacy and moral distress. The model adding direct paths from unreasonable, illegitimate tasks to approach and avoidance job crafting did not improve fit, which is consistent with the argument that unreasonable tasks matter primarily through moral distress rather than as parallel job-crafting predictors. Models that remove either unreasonable, illegitimate tasks or moral distress weaken the central theoretical claim because they collapse role-violation appraisal and ethical strain into a single stress stage. The retained model, therefore, provides the most coherent test of the proposed theory.

## 5 Discussion

The pattern of findings suggests that when doctors feel overqualified, they may not respond in a single direction. Instead, they may adjust their work in both growth-oriented and self-protective directions. This is consistent with job crafting theory (Tims et al., 2012; Wrzesniewski and Dutton, 2001) and with overqualification research, showing that perceived mismatch can motivate proactive adjustment as well as defensive regulation depending on the constraints doctors face in their roles (Fan and Shang, 2025; Shang et al., 2024).

The findings also suggest that perceived overqualification is associated with how doctors interpret their task environment, especially whether assigned duties feel reasonable for their professional role. When work includes tasks that appear poorly organized or role-incongruent, doctors may be more likely to read these assignments as disrespectful or misaligned with what should reasonably be expected from them. This interpretation matches the stress-as-offense-to-self view that illegitimate tasks are harmful because they signal disregard for the person and their role boundaries (Ma and Xie, 2024; Semmer et al., 2019). In a clinical context, this role conflict connects naturally to moral distress because moral distress arises when clinicians feel constrained from acting in line with professional and ethical standards, and this mechanism is widely documented in healthcare settings (Epstein et al., 2019; Morley et al., 2019). In practical terms, moral distress appears to be a key point at which doctors shift from trying to build resources and improve their work toward trying to limit further depletion by reducing exposure to hindering demands. This meaning is consistent with the approach–avoidance distinction in job crafting, where approach crafting requires additional effort and optimism about gains, while avoidance crafting reflects protection under strain (Rodríguez-García et al., 2024; van Leeuwen et al., 2022).

Finally, the moderation results imply that identity-related orientations can shape the extent to which this process is experienced. Calling appears to make role-incongruent demands feel more consequential because work is closely tied to purpose and moral standards, so interference with core clinical work can be felt as more personally and ethically troubling (Jiang H. et al., 2024; Kim, 2025). Professional identity appears to help contain how moral distress translates into subsequent work redesign choices, consistent with identity-based arguments that strong professional identification anchors behavior to role-consistent standards even under pressure (Cruess et al., 2014).

### 5.1 Theoretical contribution

This study makes four theoretical contributions. First, it extends perceived overqualification research by specifying a role-legitimacy pathway in clinical work. Rather than treating overqualification only as underused capability or person-job misfit, the study shows that surplus professional capability is associated with perceptions of unreasonable task assignment and moral distress among physicians.

Second, the study clarifies the distinct role of moral distress in the overqualification process. Moral distress is not used as a general strain label. It is positioned as an ethical strain state that links role-incongruent task demands to physicians’ self-regulatory responses. This distinction separates the appraisal of unreasonable work demands from the ethical strain associated with those demands and from the behavioral crafting response that may follow.

Third, the study contributes to job crafting theory by distinguishing between approach and avoidance crafting. The findings suggest that overqualification may be associated with both growth-oriented and protective work adjustment. However, when overqualification is linked to unreasonable tasks and moral distress, the pathway is associated with lower approach crafting and higher avoidance crafting.

Fourth, the study refines identity-based boundary logic. Calling appears to intensify the association between unreasonable tasks and moral distress, while professional identity appears to weaken the association between moral distress and reduced approach crafting. This shows that identity can operate both as a source of moral sensitivity and as a stabilizing professional resource.

These contributions explain why the moderated serial model is preferable to simpler alternatives. A direct model would miss the role-legitimacy mechanism. A single-mediator model would collapse task appraisal and ethical strain. A model without identity moderators would miss when the pathway becomes stronger or weaker. The contribution is therefore the specification of a clinical process linking underused expertise, role-incongruent work, moral distress, and divergent job crafting.

### 5.2 Practical implications

For senior physicians in tertiary hospital settings, these findings suggest that hospital managers can treat unreasonable, illegitimate tasks as a modifiable upstream driver of moral distress by auditing role-incongruent administrative burdens, redesigning task allocation, and strengthening role clarity so that physicians’ time and expertise are protected for clinically meaningful work. Since calling may amplify distress reactions to illegitimate demands, hospital leaders should not assume that highly called physicians are uniformly protected under constraint, and should pair purpose-oriented messaging with structural supports that reduce barriers to ethically appropriate care. Strengthening professional identity formation and professional community practices may help sustain constructive engagement when moral distress is elevated, consistent with identity-based accounts of role-consistent action under strain.

### 5.3 Limitations and future research

The study used a cross-sectional, single-source survey design in one city context, which limits causal inference and may inflate associations through shared method variance despite procedural remedies, so future research should replicate the model using longitudinal or multi-source designs and test whether changes in illegitimate task exposure precede changes in moral distress and subsequent crafting responses over time. Future studies could also examine whether professional identity buffers additional downstream outcomes of moral distress and whether calling operates as a context-dependent resource versus vulnerability under varying levels of organizational constraint, extending identity and calling perspectives in high-stakes clinical environments.

Second, all focal variables were collected from the same respondents using the same survey instrument, so common method bias remains a concern. I used procedural remedies and conducted Harman’s single-factor test and an unmeasured latent method-factor analysis. However, these diagnostics have a limited ability to detect or rule out common method bias. The study did not include a theoretically unrelated marker variable, so marker-variable correction could not be applied. Future research should collect predictor, mediator, moderator, and outcome information from different sources where possible or separate measurements across time to reduce shared-method variance more directly.

Third, the sample composition may limit generalizability. The sample was heavily male, senior, and long-tenured. Data were also collected from top-tier tertiary hospitals. Therefore, the findings should not be interpreted as representative of all physicians in China. They are most directly applicable to experienced physicians working in high-level tertiary hospital settings. Generalization to female physicians, junior physicians, physicians with shorter tenure, primary or secondary hospitals, and hospitals requires further evidence. Future research should use more balanced sampling strategies and compare the model across gender, career stage, hospital level, and region.

## Data Availability

The raw data supporting the conclusions of this article will be made available by the authors, without undue reservation.
